# Efficacy and safety of acupuncture combined with Chinese herbal medicine traditional Chinese medicine for poststroke cognitive impairment: A protocol for a systematic review and meta-analysis

**DOI:** 10.1097/MD.0000000000029019

**Published:** 2022-03-04

**Authors:** Ying Wang, Sha Guo, Meng-Lu Xiao, Hong Zhang

**Affiliations:** 1 College of Acupuncture and Moxibustion and Tuina, Chengdu University of Traditional Chinese Medicine, Sichuan, China.

**Keywords:** acupuncture, poststroke cognitive impairment, protocol, systematic review, traditional Chinese medicine

## Abstract

**Background:**

Poststroke cognitive impairment (PSCI) is often secondary to poststroke, which is common in stroke patients, induced difficulty in rehabilitation and seriously affects their quality of life. Currently, effective treatments are still limited. Researches show that acupuncture combined with traditional Chinese medicine (TCM) has a good effect on PSCI. However, there is no consistent conclusion at present. Therefore, THE purpose of this study is to assess the effectiveness and safety of acupuncture combined with TCM in the treatment of PSCI.

**Methods:**

We will search the following databases from inception to January, 2022: the Cochrane Library, PubMed, Embase, Medline, Web of Science, China National Knowledge Infrastructure, China Biology Medicine, Wan Fang data, and the Chinese Science and Technology Periodical Database. All randomized controlled trials eligible for acupuncture combined with TCM for PSCI will be included in this study. Study selection, data extraction, and quality assessment will be performed by 2 reviewers independently. Bias risk assessment and data synthesis will be performed using the Review Manager software (RevMan, version 5.3) and R (version 3.6.1) software.

**Results:**

We will synthesize the current studies to evaluate the effectiveness and safety of acupuncture combined with TCM in the treatment of PSCI.

**Conclusion:**

The systematic review will provide a new paradigm for acupuncture combined with TCM in the intervention of PSCI, and further provide scientific evidence for the efficacy and safety of acupuncture combined with TCM in the treatment of PSCI.

**Trial registration number:**

INPLASY202220062.

## 1 Introduction

Poststroke cognitive impairment (PSCI) is one of the most common complications of stroke, which is characterized by advanced brain dysfunction such as attention, memory, executive ability, language, and perceptual motor function in stroke patients.^[1]^ It directly affects the compliance with rehabilitation and treatment outcome of stroke patients, and can predict functional prognosis and survival rate of stroke patients.^[2]^ Epidemiological studies have shown that the global prevalence of PSCI ranges from 20% to 80% based on different diagnostic criteria, ethnicity.^[3–5]^ PSCI has become a serious public health problem, bringing heavy burden to patients’ families and society.^[6]^ Importantly, the exact treatment used for PSCI is unclear.

Currently, the treatment methods mainly focus on western medicine and cognitive rehabilitation. Both approaches are used in clinical practice, mainly to improve their symptoms. Although western medicine treatment has certain potential, it also has potential side effects.^[7]^ And there is insufficient evidence to support the improvement of individual cognitive function with cognitive rehabilitation.^[8]^

Notably, numerous clinical studies have found that acupuncture combined with oral administration of traditional Chinese medicine (TCM) is effective in the treatment of PSCI.^[9,10]^ However, the quality of these literatures vary, resulting in inconsistent results among studies. Therefore, the evidence concluding the efficacy of acupuncture combined with TCM in the treatment of PSCI is not sufficient. Therefore, this protocol is designed to verify the efficacy and safety of acupuncture combined with TCM in the treatment of PSCI. In this study, a randomized controlled trial (RCT) of acupuncture combined with TCM for PSCI will be systematically evaluated using an evidence-based approach to provide a basis for the future clinical use of acupuncture combined with TCM in the treatment of PSCI.

## 2 Methods

The protocol of this systematic review will be reported following the Preferred Reporting Items for Systematic Review and Meta-analysis Protocol (PRISMA-P) 2015^[11]^ guidelines. Our review protocol was registered with INPLASY (number: INPLASY202220062).

### 2.1 Eligibility criteria

#### 2.1.1 Types of studies

All clinical RCTs about acupuncture combined with TCM for PSCI will be included in this study. Non-RCTs, a series of case reports, observational studies, animal studies, letters, editorials, conferences, and comments will be excluded. Language will be limited to Chinese and English.

#### 2.1.2 Types of participants

Only patients with a clinical diagnosis of PSCI will be considered for this study, and there are no restrictions on diagnostic criteria, age, sex, race, gender, economic status, or education level.

#### 2.1.3 Types of interventions and comparators

All patients with PSCI included in this study received conventional treatment. Patients in the intervention group was treated with acupuncture (including electroacupuncture, scalp acupuncture, and other treatment methods) combined with TCM. The control group was treated with conventional western treatment.

#### 2.1.4 Types of outcomes

##### 2.1.4.1 Primary outcomes

The primary outcome of the study is cognitive function, and will be assessed by the Montreal Cognitive Assessment scale and the Mini-Mental State Examination.

##### 2.1.4.2 Secondary outcomes

The secondary outcomes will include as follow:

1. Attentional performance: Test of Attentional Performance;
2. Memory: Wechsler Memory Scale;
3. Quality of life: Short Form Survey Instrument, Fugl-Meyer Motor Assessment, Activities of daily living;
4. Adverse events related to acupuncture combined with TCM treatment (e.g., stuck needle, pain or discomfort, gastrointestinal reaction, hepatic damage).

### 2.2 Search strategy

The Cochrane Library, PubMed, Embase, Medline, Web of Science, China National Knowledge Infrastructure, Wan fang, Chinese Scientific Journal Database, and Chinese Biomedical Literature Database will be searched for RCTs of acupuncture combined with TCM for PSCI. To avoid omissions, we also searched special databases and international experiment registration websites (e.g., the World Health Organization International Registry of Clinical Trials). All literature published in Chinese or English before January, 2022 will be included.

Taking PubMed as an example, the detailed search process is shown in Table 1. The similar search strategy will be applied to other databases, and the terms will be modified as the characteristics different databases needed.

**Table 1:** Search strategy for PubMed database.

Number	Terms
#1	Post-stroke cognitive impairment[all field]
#2	PSCI[all field]
#3	Post-stroke dementia[all field]
#4	#1 OR #2-3
#5	Strokes[all field]
#6	Cerebrovascular Accident[all field]
#7	Cerebrovascular Apoplexy[all field]
#8	Cerebral haemorrhage[all field]
#9	Cerebral ischaemia[all field]
#10	Cerebral infarct[all field]
#11	Brain infarct[all field]
#12	Brain haemorrhage[all field]
#13	#5 OR #6-12
#14	Cognitive Dysfunctions[all field]
#15	Cognitive Impairment[all field]
#16	Cognitive Impairments[all field]
#17	Cognitive Decline[all field]
#18	Cognition[all field]
#19	#14 OR #15-18
#20	Acupuncture[all field]
#21	Acupuncture treatment[all field]
#22	Acupoint[all field]
#23	Needling[all field]
#24	Intradermal needling[all field]
#25	Electro acupuncture[all field]
#26	Fire needling[all field]
#27	Scalp acupuncture[all field]
#28	Catgut embedding[all field]
#29	Ear acupuncture[all field]
#30	Auricular acupuncture[all field]
#31	#20 or #21-30
#32	Traditional Chinese Medicine[all field]
#33	Chinese Medicine[all field]
#34	Chinese Herbs[all field]
#35	Chinese Herbal Drugs[all field]
#36	Chinese herbal[all field]
#37	#32 or #33-36
#38	Randomized controlled trial[all field]
#39	Random allocation[all field]
#40	Randomized[all field]
#41	Random[all field]
#42	Controlled clinical trial[all field]
#43	Clinical Trial[all field]
#44	Trial[all field]
#45	#38 OR #39-44
#46	#4 And #13 And #19 And #31 And #37 And #45

### 2.3 Data collection and analysis

**Figure 1:**
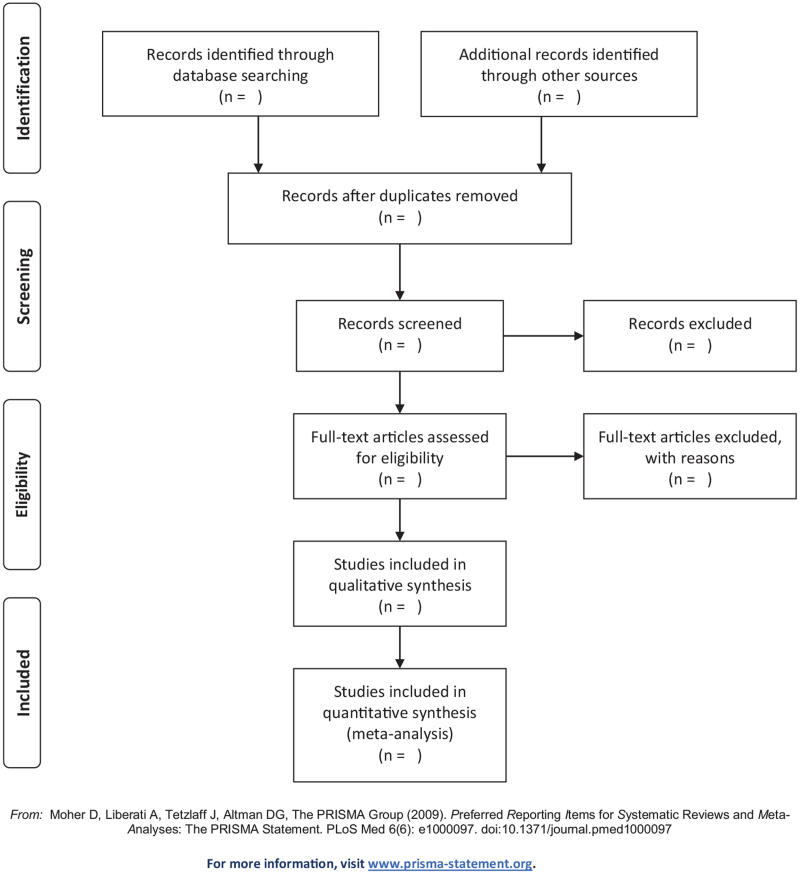
Flow diagram of studies selection.

All retrieved literatures will be managed using EndNote X9 (Thomson Reuters, CA, USA) and repeated records will be filtered. Frist, 2 reviewers (YW and SG) will independently examine and evaluate all literatures according to the title and abstract based on the pre-set inclusion criteria. Second, download the preliminary selection articles, and further independently review the full text. Finally, 2 reviewers will cross-examine the included studies. Differences will be resolved through discussion or consensus with third-party reviewers (MLX). The program selected for the study will be carried out according to the PRISMA flow chart (Fig. 1).

#### 2.3.1 Data extraction and management

Two reviewers (YW and SG) will independently extract data with a pre-defined data extraction form which include the following information: general characteristics (title, author, year of publication, country, and journal), characteristics of participants (baseline data, PSCI diagnostic criteria, exclusion criteria, gender, age), other characteristics (study design, sample size, interventions in treatment group and control group), and primary and secondary outcomes (Test of Attentional Performance, Wechsler Memory Scale, Fugl-Meyer Motor Assessment, Activities of daily living, Short Form Survey Instrument, adverse events for acupuncture combined with TCM treatment). When above data in an article is incomplete, we will contact the author by email to request additional information, and the differences will be settled through consensus or discussion with a third reviewer (MLX).

#### 2.3.2 Assessment of risk of bias

The Cochrane Collaboration Tool with risk of bias will be used to conduct the quality of included articles by 2 independent assessment reviewers (YW and SG). The following 7 dimensions will be assessed:

(1) random sequence generation;
(2) allocation concealment;
(3) blinding of the participants and personnel;
(4) blinding of outcome assessment;
(5) incomplete outcome data;
(6) selective reporting;
(7) other sources of bias.

The risk of bias will be divided into 3 levels: low risk, high risk, and unclear risk. All stages of the quality assessment process are independently performed by 2 reviewers (YW and SG). Any discrepancies will be resolved through discussion or consensus with third-party reviewers.

#### 2.3.3 Measures of treatment effect

The continuous outcomes will be recorded as mean difference or standard mean difference and 95% confidence interval. For dichotomous data, the risk ratio with 95% confidence intervals will be used to evaluate the treatment effect.

#### 2.3.4 Dealing with missing data

When the article data are missing or incomplete, we will try to contact the first author or the corresponding author to obtain complete information. If accurate data are not available, we will analyze the available data.

#### 2.3.5 Assessment of heterogeneity

We will use I^2^ value of the chi-squared test to detect the heterogeneity of included studies. If I^2^ ≤ 50%, it means acceptable homogeneity. Otherwise, if I^2^ > 50%, it means significant heterogeneity.

#### 2.3.6 Data synthesis

A data synthesis will use RevMan 5.3 (The Nordic Cochrane Centre for The Cochrane Collaboration, Copenhagen, Denmark). We will use a fixed-effects model if there is no statistical heterogeneity among studies, and perform meta-analysis. Otherwise, we will use a random-effects model if there is statistical heterogeneity. If there is significant heterogeneity, we will conduct subgroup analysis or sensitivity analysis.

#### 2.3.7 Subgroup analysis

If necessary, subgroup analysis will be performed to detect the source of heterogeneity. Type of acupuncture stimulation, different TCM decoctions, subject (age, gender), and duration of treatment will be considered.

#### 2.3.8 Sensitivity analysis

In order to check the robustness of the results, we will conduct a sensitivity analysis to assess the impact of studies with a high risk of bias.

## 3 Discussion

Cognitive impairment in stroke patients seems to have a negative impact on their recovery of motor function, language function, and activities of daily living.^[12]^ This makes the overall rehabilitation of PSCI patients more difficult and hinders their reintegration into their families and society.^[13,14]^ Almalki et al^[15]^ showed that cognitive function could be used as a predictor of functional outcome in stroke patients. Therefore, it is necessary to find thorough and effective treatments.

The therapy of acupuncture combined with TCM, as a common clinical adjuvant therapy, has the advantages of simple operation, few side effects, and enhanced efficacy. Clinical studies have found that acupuncture combined with TCM is effective for various diseases, including dementia.^[16,17]^ Furthermore, acupuncture combined with TCM is also effective in the treatment of PSCI.^[10]^ The combined use of acupuncture and TCM can provide comprehensive management of poststroke symptoms in stroke patients, thus improving their cognitive function.^[18]^ Based on this, the current study systematically reviewed the efficacy and safety of acupuncture combined with TCM in the treatment of PSCI, in order to provide a strong scientific basis for clinical application.

In addition, there are some limitations in this study. Firstly, different types of acupuncture, and TCM decoctions will have different efficacy, which might be caused bias in the results. Moreover, only articles in Chinese and English will be searched, which may lead to some publication bias.

### 3.1 Amendments

We will update our protocol if there are any changes during the research.

## Author contributions

**Conceptualization:** Ying Wang, Hong Zhang.

**Data curation:** Ying Wang, Sha Guo.

**Formal analysis:** Ying Wang, Sha Guo, Meng-Lu Xiao.

**Funding acquisition:** Hong Zhang.

**Investigation:** Ying Wang, Meng-Lu Xiao.

**Methodology:** Sha Guo, Meng-Lu Xiao.

**Project administration:** Ying Wang.

**Resources:** Ying Wang.

**Software:** Ying Wang.

**Supervision:** Hong Zhang.

**Validation:** Ying Wang.

**Visualization:** Ying Wang.

**Writing – original draft:** Ying Wang.

**Writing – review & editing:** Ying Wang, Sha Guo, Meng-Lu Xiao, Hong Zhang.

Zhang Hong is the guarantor of this review. All the authors approved the publication of the protocol.
